# Supplementary material for: High diversity and variability of pipolins among a wide range of pathogenic Escherichia coli strains
Source: Sci Rep. 2020 Jul 27;10:12452. doi: 10.1038/s41598-020-69356-6 (PMC7385651; doi:10.1038/s41598-020-69356-6)
Supplement: Supplementary file 1 — Supplementary Figures [file 41598_2020_69356_MOESM1_ESM.pdf]

**High diversity and variability of pipolins among a wide range of pathogenic *Escherichia coli* strains**

Saskia-Camille Flament-Simon[1*], María de Toro[2*], Liubov Chuprikova[4*], Miguel Blanco[1], Juan Moreno-González[3], Margarita Salas[3‡], Jorge Blanco[1], and Modesto Redrejo-Rodríguez[3,4#]

[1] Laboratorio de Referencia de *E. coli* (LREC), Departamento de Microbiología y Parasitología, Facultad de Veterinaria, Universidad de Santiago de Compostela (USC), Lugo, 27002,Spain.

[2] Plataforma de Genómica y Bioinformática, CIBIR (Centro de Investigación Biomédica de La Rioja), Logroño, 26006 La Rioja, Spain.

[3] Centro de Biología Molecular Severo Ochoa (Consejo Superior de Investigaciones Científicas-Universidad Autónoma de Madrid), 28049, Madrid, Spain.

[4] Departamento de Bioquímica, Universidad Autónoma de Madrid (UAM), Instituto de Investigaciones Biomédicas "Alberto Sols" CSIC-UAM, 28029, Madrid, Spain.

*Equal contribution

‡Deceased

#Correspondence to modesto.redrejo@uam.es

# SUPPLEMENTARY INFORMATION

## Supplementary Tables

**Table S1 (XLS file). Characterization of pipolin-harboring LREC strains.**
Compilation of main features determined for LREC strains. Biosample and Enterobase Uberstrain reference IDs are also indicated. D*: *Does not match with PCR data. N.D. : non-detected.* Virulence factors detected by PCR screening and in silico analysis are detailed.

**Table S2 (XLS file). Genbank genomes carrying pipolins.**
References for genomes and biosamples as well as main features from Enterobase [21] are indicated.
*CRISPR/Cas cassettes and Integrons were surveyed as indicated in Methods.
**Integrity of detected integrons were analyzed with IntegronFinder [56] as indicated in Materials and Methods. C, complete; In0, integron lacking attC site; CALIN, integron lacking functional integrase gene.
***When available, PubMed ID of strain reporting publication

| Gene | Pipolins (%) | Annotation | UniProtKB HHPred best hit | eggNOG Description | KEGG KO | KEGG Description |
|---|---|---|---|---|---|---|
| pipolB | 92 (100%) | Primer-independent DNA polymerase PolB | P03680 | | | |
| xerC_2 | 90 (98%) | Tyrosine recombinase XerC | P0A8P8 | Belongs to the 'phage' integrase family | | |
| group_1 | 87 (95%) | Uracil-DNA glycosylase | Q96YD0 | | | |
| group_6 | 86 (93%) | hypothetical protein | | | | |
| xerC_1 | 84 (85%) | Tyrosine recombinase XerC | P03700 | Belongs to the 'phage' integrase family | | |
| hisF | 78 (83%) | Type I restriction modification system methyltransferase (hsdM) | Q5M500 | HsdM N-terminal domain | K03427 | hsdM; type I restriction enzyme M protein [EC:2.1.1.72] |
| group_16 | 76 (82%) | metallohydrolase | Q57587 | Metal-dependent hydrolase | K07043 | uncharacterized protein |
| group_18 | 75 (82%) | hypothetical protein | | | | |
| group_5 | 75 (82%) | Type I site-specific deoxyribonuclease (hsdR) | P10486 | Type I restriction enzyme R protein N terminus (HSDR_N) | K01153 | hsdR; type I restriction enzyme, R subunit [EC:3.1.21.3] |
| group_10 | 75 (82%) | Protein of unknown function (DUF2787) | | Protein of unknown function (DUF2787) | | |
| group_13 | 74 (80%) | hypothetical protein | | Protein of unknown function (DUF726) | | |
| group_11 | 73 (79%) | hypothetical protein | | | | |
| group_24 | 73 (79%) | hypothetical protein | | | | |
| group_52 | 73 (79%) | Excisionase | A6T888 | | | |
| group_3 | 64 (70%) | hypothetical protein | | | | |
| group_8 | 59 (64%) | hypothetical protein | | | | |
| group_19 | 56 (61%) | WYL domain | A0A4Y3NDN0 | transcriptional regulator | | |
| group_58 | 53 (58%) | Znf/thioredoxin_put domain-containing protein | Q9A679 | | | |
| group_28 | 41 (45%) | Uncharacterized protein family (UPF0149) | P28366 | Uncharacterised protein family (UPF0149) | K07039 | uncharacterized protein |
| group_17 | 38 (41%) | IS1 family transposase IS1A | | cog cog3677 | | |
| group_23 | 31 (34%) | PD-(D/E)XK nuclease superfamily | | PD-(D/E)XK nuclease superfamily | | |
| group_31 | 30 (33%) | Restriction endonuclease | A0A0J9X157 | Restriction endonuclease | | |
| group_15 | 30 (33%) | IS1 family transposase IS1X2 | | cog cog1662 | K07480 | insertion element IS1 protein InsB |
| group_9 | 29 (32%) | Protein of unknown function (DUF4011) | | Protein of unknown function (DUF4011) | | |

| | | | | | | |
|---|---|---|---|---|---|---|
| group_34 | 24 (26%) | hypothetical protein | | type I restriction enzyme, R | | |
| group_53 | 23 (25%) | Protein of unknown function DUF262 | | Protein of unknown function (DUF1524) | | |
| group_14 | 22 (24%) | Uncharacterized protein family (UPF0149) | | | | |
| group_20 | 22 (24%) | WYL-domain containing protein | A0A4Y3NDN0 | | | |
| group_39 | 22 (24%) | Protein of unknown function DUF262 | | Protein of unknown function (DUF1524) | | |
| group_40 | 22 (24%) | hypothetical protein | | | | |
| group_25 | 22 (24%) | Protein of unknown function DUF262 | | Protein of unknown function (DUF1524) | | |
| group_55 | 21 (23%) | Protein of unknown function DUF262 | | Protein of unknown function DUF262 | | |
| group_32 | 19 (21%) | Znf/thioredoxin_put domain-containing protein | Q9A679 | | | |
| group_27 | 16 (17%) | Type I restriction modification enzyme | Q8R9Q6 | Type I restriction modification DNA specificity domain | K01154 | hsdS; type I restriction enzyme, S subunit [EC:3.1.21.3] |
| group_29 | 16 (17%) | IS3 family transposase ISEam1 | A0A0G3QIX7 | Transposase | K07483 | transposase |
| insK | 78 (16%) | IS3 family transposase ISEc14 | T0PD67 | silverDB | | |

**Table S3. Functional characterization of the most common pipolin genes.**

Annotation of genes from Roary shell-genome (present in more than 15% of pipolins) is indicated. Functional groups in eggNOG and KEGG databases, as well as HHPred searches were also performed for a detailed functional characterization.

## Supplementary Figures

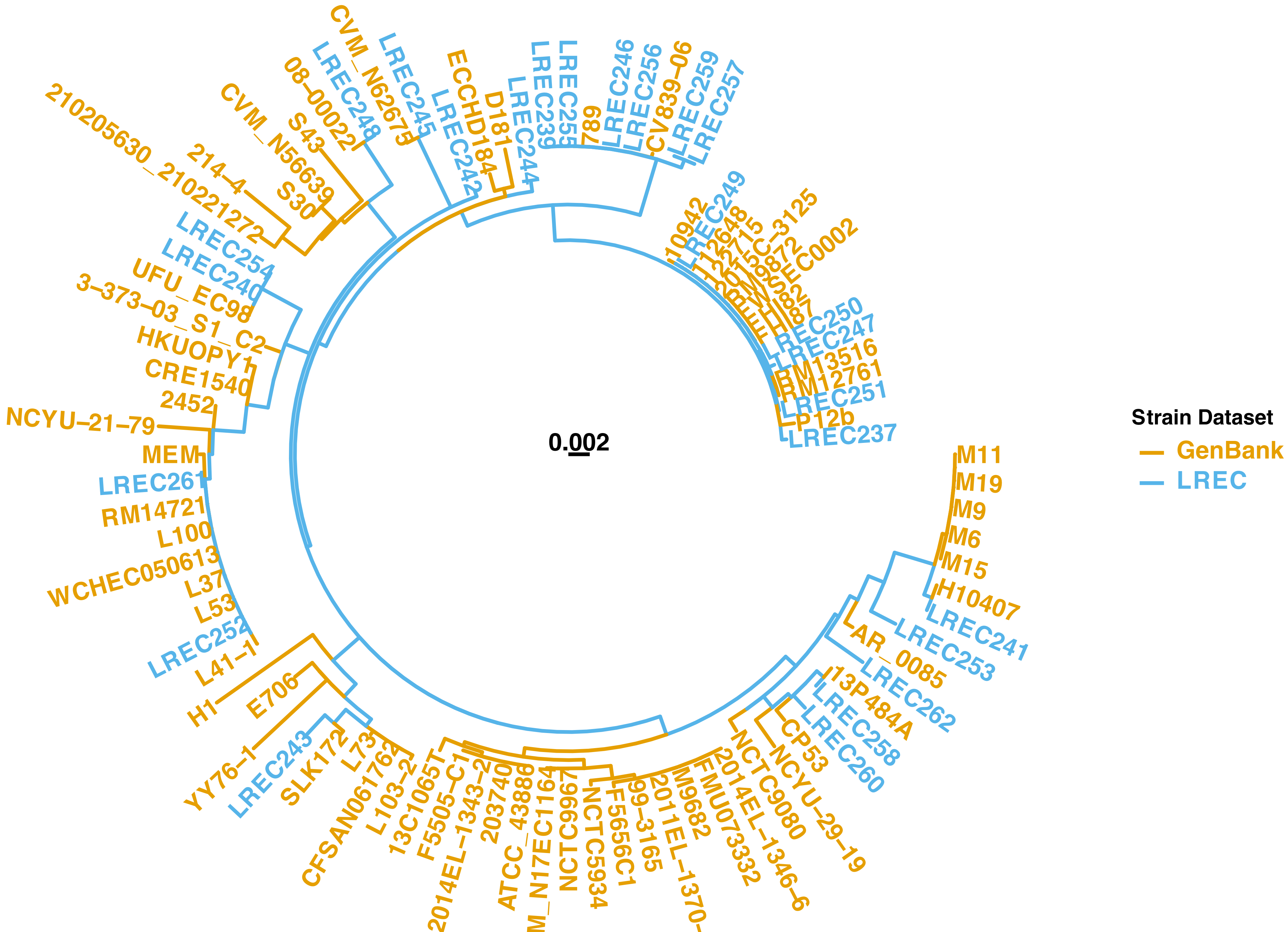

0.002
Strain Dataset
GenBank
LREC
210205630_210221272
214-4
S30
CVM_N56639
S43
08-00022
LREC248
CVM_N62675
LREC245
LREC242
ECCHD184
D181
LREC244
LREC239
LREC255
789
LREC246
LREC256
CV839-06
LREC259
LREC257
10942
LREC249
EC0002
LREC250
LREC247
RM13516
RM12761
LREC251
P12b
LREC237
M11
M19
M9
M6
M15
H10407
LREC241
LREC253
AR_0085
LREC262
13P484A
LREC258
LREC260
CP53
NCYU-29-19
NCTC9080
2014EL-1346-6
FMU073332
M9682
2011EL-1370-2
99-3165
F5656C1
NCTC5934
NCTC9967
CVM_N17EC1164
ATCC_43886
203740
2014EL-1343-2
F5505-C1
13C1065T
L103-2
CFSAN061762
L73
SLK172
LREC243
YY76-1
E706
H1
L41-1
LREC252
L53
L37
WCHEC050613
L100
RM14721
LREC261
MEM
NCYU-21-79
2452
CRE1540
HKUOPY1
3-373-03_S1_C2
UFU_EC98
LREC240
LREC254


Figure S1

**Figure S1. Phylogeny of piPolB genes from analyzed pipolins.**
Nucleotide sequence of piPolB genes from new pipolins in LREC strains (cyan) and those retrieved from GenBank (orange) were aligned using Prank codon aware option and then used for maximum-likelihood phylogeny reconstruction with IQtree. Modelfinder Best-fit model was K3Pu+F+R2.

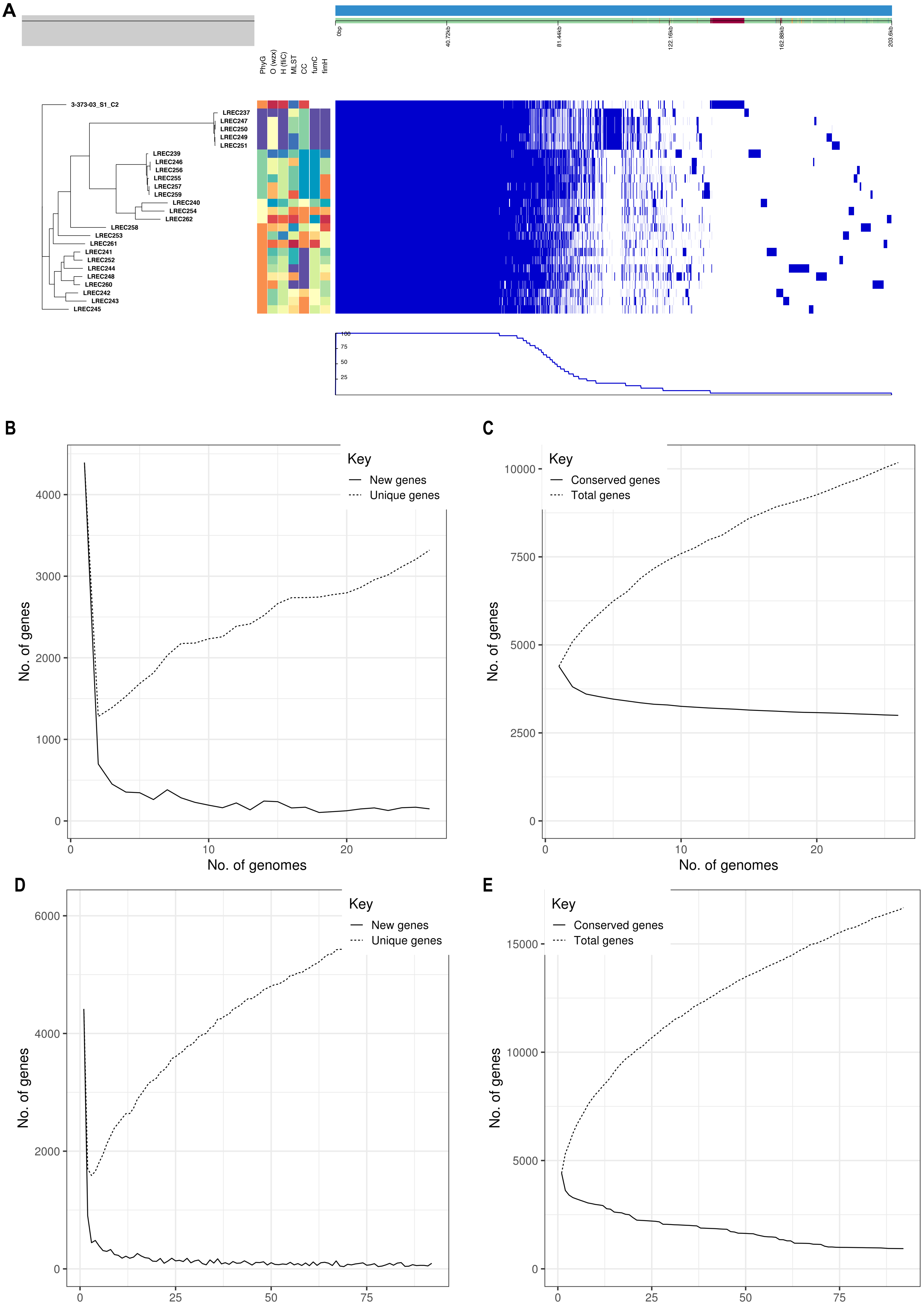
A
B
C
D
E
PhyG
O (wzx)
H (fliC)
MLST
CC
fumC
fimH
0bp
40.72kb
81.44kb
122.16kb
162.88kb
203.6kb
3-373-03_S1_C2
LREC237
LREC247
LREC250
LREC249
LREC251
LREC239
LREC246
LREC256
LREC255
LREC257
LREC259
LREC240
LREC254
LREC262
LREC258
LREC253
LREC261
LREC241
LREC252
LREC244
LREC248
LREC260
LREC242
LREC243
LREC245
Key
New genes
Unique genes
Conserved genes
Total genes
No. of genes
No. of genomes

Figure S2

**Figure S2. Roary pangenome analysis of pipolins-harboring *E. coli* strains.**
A. Combined layout of hierarchical clustering of gene presence/absence for LREC *E. coli* genomes (left), along with color-coded markers (middle) and chromosome genetic structure (right). Representation of Roary output was rendered at Phandango website [67]. Lower panels show the accumulation of new vs. unique genes per isolate (B and D) and conserved vs. total genes per isolate (C and E) in the new (LREC, B and C) and all (LREC+GenBank, D and E) *E. coli* strains.

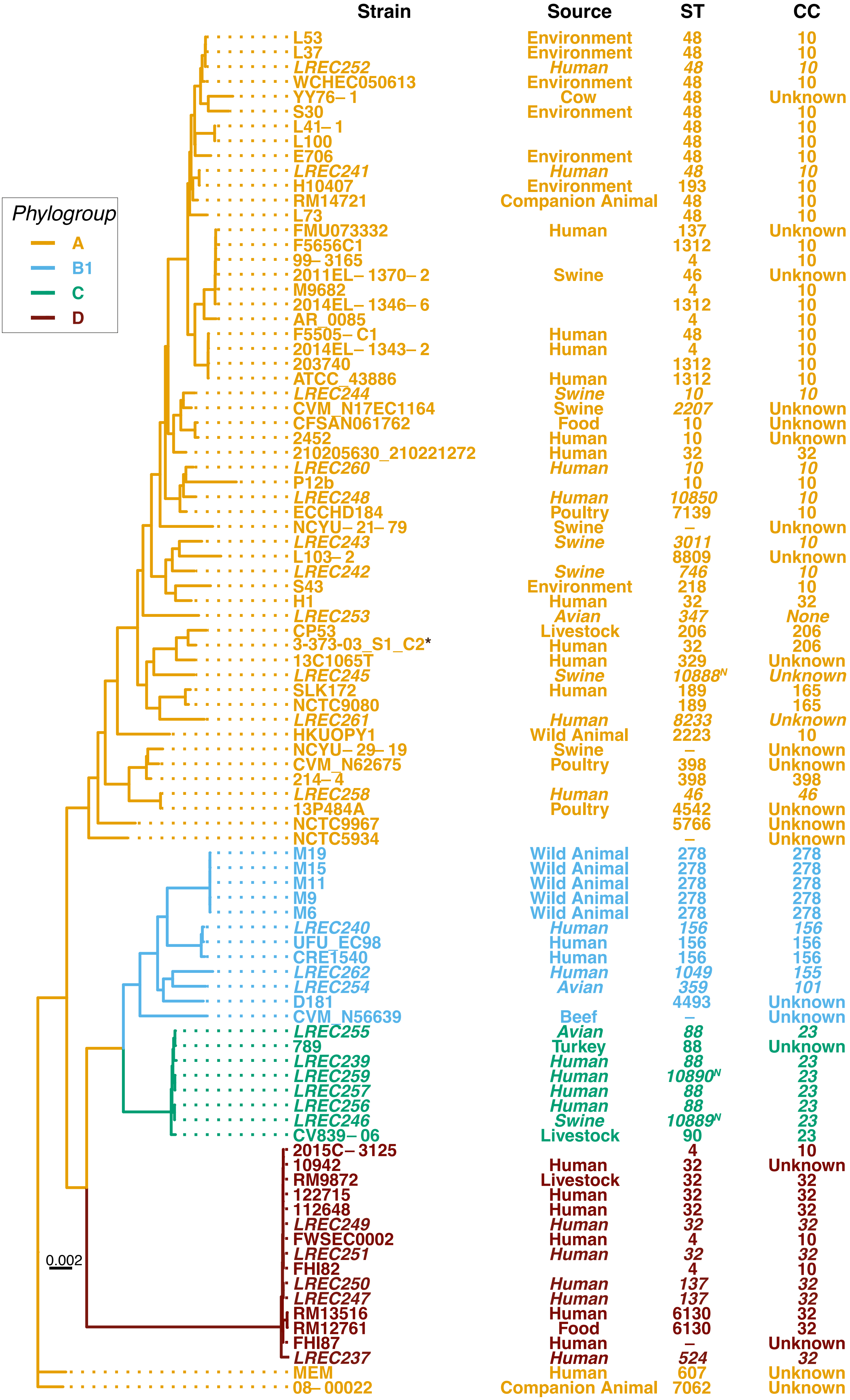
Strain Source ST CC
Phylogroup
A
B1
C
D
0.002
L53 Environment 48 10
L37 Environment 48 10
LREC252 Human 48 10
WCHEC050613 Environment 48 10
YY76–1 Cow 48 Unknown
S30 Environment 48 10
L41–1 48 10
L100 48 10
E706 Environment 48 10
LREC241 Human 48 10
H10407 Environment 193 10
RM14721 Companion Animal 48 10
L73 48 10
FMU073332 Human 137 Unknown
F5656C1 1312 10
99–3165 4 10
2011EL–1370–2 Swine 46 Unknown
M9682 4 10
2014EL–1346–6 1312 10
AR_0085 4 10
F5505–C1 Human 48 10
2014EL–1343–2 Human 4 10
203740 1312 10
ATCC_43886 Human 1312 10
LREC244 Swine 10 10
CVM_N17EC1164 Swine 2207 Unknown
CFSAN061762 Food 10 Unknown
2452 Human 10 Unknown
210205630_210221272 Human 32 32
LREC260 Human 10 10
P12b 10 10
LREC248 Human 10850 10
ECCHD184 Poultry 7139 10
NCYU–21–79 Swine – Unknown
LREC243 Swine 3011 10
L103–2 8809 Unknown
LREC242 Swine 746 10
S43 Environment 218 10
H1 Human 32 32
LREC253 Avian 347 None
CP53 Livestock 206 206
3-373-03_S1_C2* Human 32 206
13C1065T Human 329 Unknown
LREC245 Swine 10888$^{N}$ Unknown
SLK172 Human 189 165
NCTC9080 189 165
LREC261 Human 8233 Unknown
HKUOPY1 Wild Animal 2223 10
NCYU–29–19 Swine – Unknown
CVM_N62675 Poultry 398 Unknown
214–4 398 398
LREC258 Human 46 46
13P484A Poultry 4542 Unknown
NCTC9967 5766 Unknown
NCTC5934 – Unknown
M19 Wild Animal 278 278
M15 Wild Animal 278 278
M11 Wild Animal 278 278
M9 Wild Animal 278 278
M6 Wild Animal 278 278
LREC240 Human 156 156
UFU_EC98 Human 156 156
CRE1540 Human 156 156
LREC262 Human 1049 155
LREC254 Avian 359 101
D181 4493 Unknown
CVM_N56639 Beef – Unknown
LREC255 Avian 88 23
789 Turkey 88 Unknown
LREC239 Human 88 23
LREC259 Human 10890$^{N}$ 23
LREC257 Human 88 23
LREC256 Human 88 23
LREC246 Swine 10889$^{N}$ 23
CV839–06 Livestock 90 23
2015C–3125 4 10
10942 Human 32 Unknown
RM9872 Livestock 32 32
122715 Human 32 32
112648 Human 32 32
LREC249 Human 32 32
FWSEC0002 Human 4 10
LREC251 Human 32 32
FHI82 4 10
LREC250 Human 137 32
LREC247 Human 137 32
RM13516 Human 6130 32
RM12761 Food 6130 32
FHI87 Human – Unknown
LREC237 Human 524 32
MEM Human 607 Unknown
08–00022 Companion Animal 7062 Unknown

Figure S3

**Figure S3. Maximum-likelihood phylogeny of pipolins-harboring *E. coli* strains.** Codon aware core-genome alignment from Roary was used for the phylogeny reconstruction using IQtree. The best-fit model was GTR+F+R7 (according to ModelFinder). Scale bar indicates the substitution rate per site. The main features retrieved from Enterobase are indicated on the right: source, multilocus sequence type (ST) and clonal complexes (CC). Strains are colored based on the phylogenetic groups, with LREC strains highlighted in italics. Reference strain 3-373-03_S1_C2 is highlighted with a black asterisk (*).

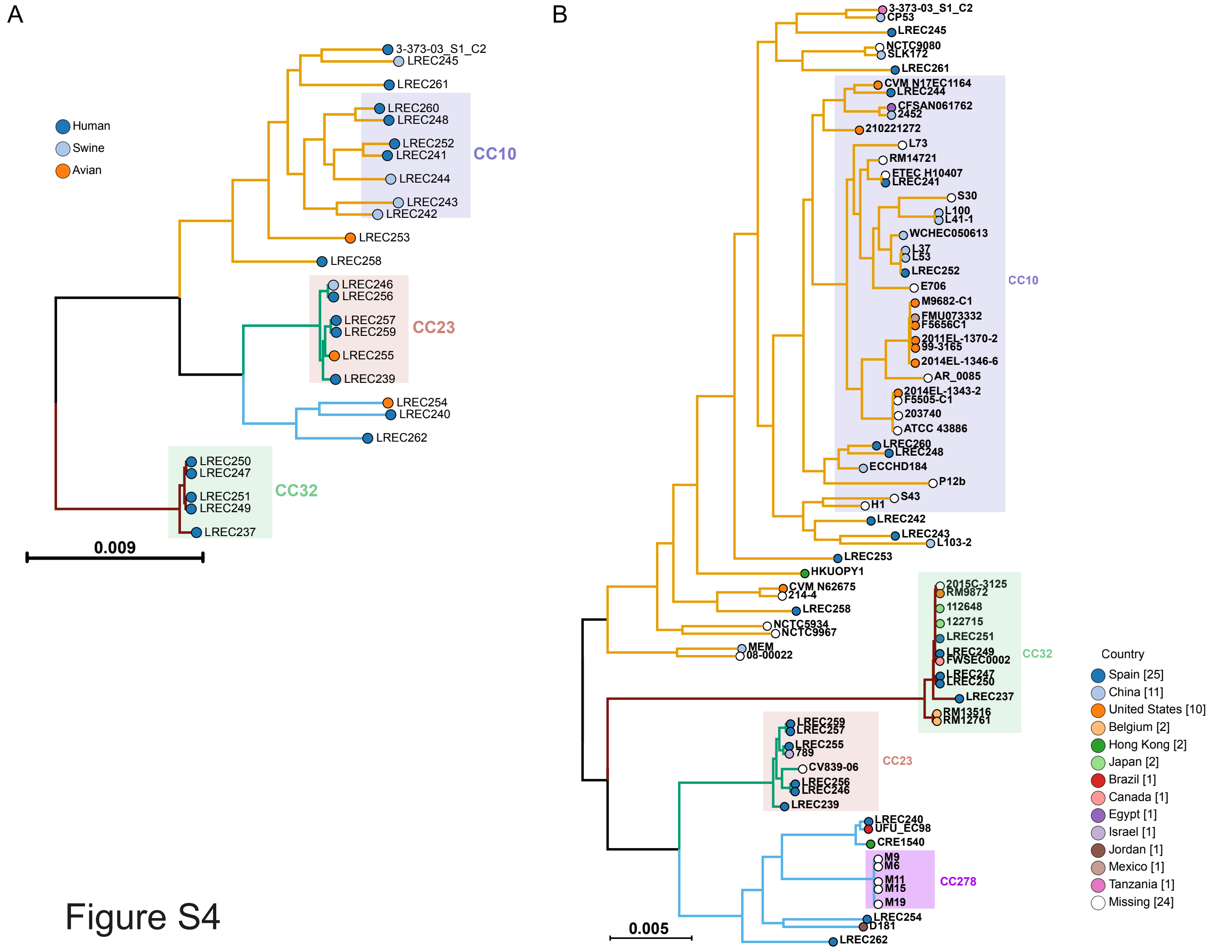

A
3-373-03_S1_C2
LREC245
LREC261
LREC260
LREC248
LREC252
LREC241
LREC244
LREC243
LREC242
CC10
LREC253
LREC258
LREC246
LREC256
LREC257
LREC259
LREC255
LREC239
CC23
LREC254
LREC240
LREC262
LREC250
LREC247
LREC251
LREC249
LREC237
CC32
Human
Swine
Avian
0.009
B
3-373-03_S1_C2
CP53
LREC245
NCTC9080
SLK172
LREC261
CVM N17EC1164
LREC244
CFSAN061762
2452
210221272
L73
RM14721
ETEC H10407
LREC241
S30
L100
L41-1
WCHEC050613
L37
L53
LREC252
E706
M9682-C1
FMU073332
F5656C1
2011EL-1370-2
99-3165
2014EL-1346-6
AR_0085
2014EL-1343-2
F5505-C1
203740
ATCC 43886
LREC260
LREC248
ECCHD184
P12b
S43
H1
LREC242
LREC243
L103-2
LREC253
HKUOPY1
CVM N62675
214-4
LREC258
NCTC5934
NCTC9967
MEM
08-00022
CC10
2015C-3125
RM9872
112648
122715
LREC251
LREC249
FWSEC0002
LREC247
LREC250
LREC237
RM13516
RM12761
CC32
LREC259
LREC257
LREC255
789
CV839-06
LREC256
LREC246
LREC239
CC23
LREC240
UFU_EC98
CRE1540
M9
M6
M11
M15
M19
CC278
LREC254
D181
LREC262
0.005
Country
Spain [25]
China [11]
United States [10]
Belgium [2]
Hong Kong [2]
Japan [2]
Brazil [1]
Canada [1]
Egypt [1]
Israel [1]
Jordan [1]
Mexico [1]
Tanzania [1]
Missing [24]


Figure S4

**Figure S4. Single Nucleotide Polymorphism (SNP) trees of the pipolin-harboring strains.**

A. LREC strains' SNP-based tree. Previously described pipolin-harboring isolate 3-373-03_S1_C2 was included as the reference genome. The SNP-tree was performed with EnteroBase [21] default parameters (min. 95% sites present) and included 132985 variant sites. Source and main clonal complexes are also indicated. B. SNP-based tree of the 58 pipolin-harboring strains available from Enterobase and the 25 LREC pipolin-harboring strains performed with EnteroBase. The tree included 170702 variant sites. The figure also includes the country of isolation, sequence types (ST) and clonal complexes (CC). Clade branches were colored by phylogenetic groups as in previous figures.

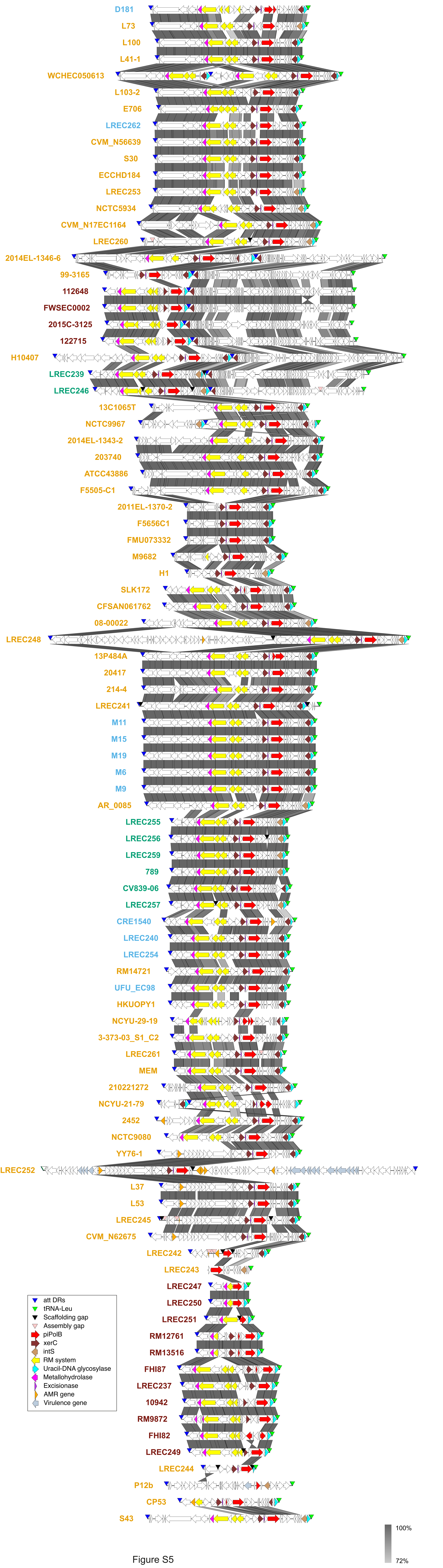
D181
L73
L100
L41-1
WCHEC050613
L103-2
E706
LREC262
CVM_N56639
S30
ECCHD184
LREC253
NCTC5934
CVM_N17EC1164
LREC260
2014EL-1346-6
99-3165
112648
FWSEC0002
2015C-3125
122715
H10407
LREC239
LREC246
13C1065T
NCTC9967
2014EL-1343-2
203740
ATCC43886
F5505-C1
2011EL-1370-2
F5656C1
FMU073332
M9682
H1
SLK172
CFSAN061762
08-00022
LREC248
13P484A
20417
214-4
LREC241
M11
M15
M19
M6
M9
AR_0085
LREC255
LREC256
LREC259
789
CV839-06
LREC257
CRE1540
LREC240
LREC254
RM14721
UFU_EC98
HKUOPY1
NCYU-29-19
3-373-03_S1_C2
LREC261
MEM
210221272
NCYU-21-79
2452
NCTC9080
YY76-1
LREC252
L37
L53
LREC245
CVM_N62675
LREC242
LREC243
LREC247
LREC250
LREC251
RM12761
RM13516
FHI87
LREC237
10942
RM9872
FHI82
LREC249
LREC244
P12b
CP53
S43
att DRs
tRNA-Leu
Scaffolding gap
Assembly gap
piPolB
xerC
intS
RM system
Uracil-DNA glycosylase
Metallohydrolase
Excisionase
AMR gene
Virulence gene
100%
72%

Figure S5

**Figure S5. Genetic structure of pipolins.**
Protein-coding genes are represented by arrows, indicating the direction of transcription, and colored as indicated in the legend. The image was generated by EasyFig software and re-annotated pipolins sorted according to the hierarchical clustering of the gene presence/absence. The greyscale on the right reflects the percent of amino acid identity between pairs of sequences. Names of pipolin-carrying strains are colored based in the phylogroups as in Figure S3.

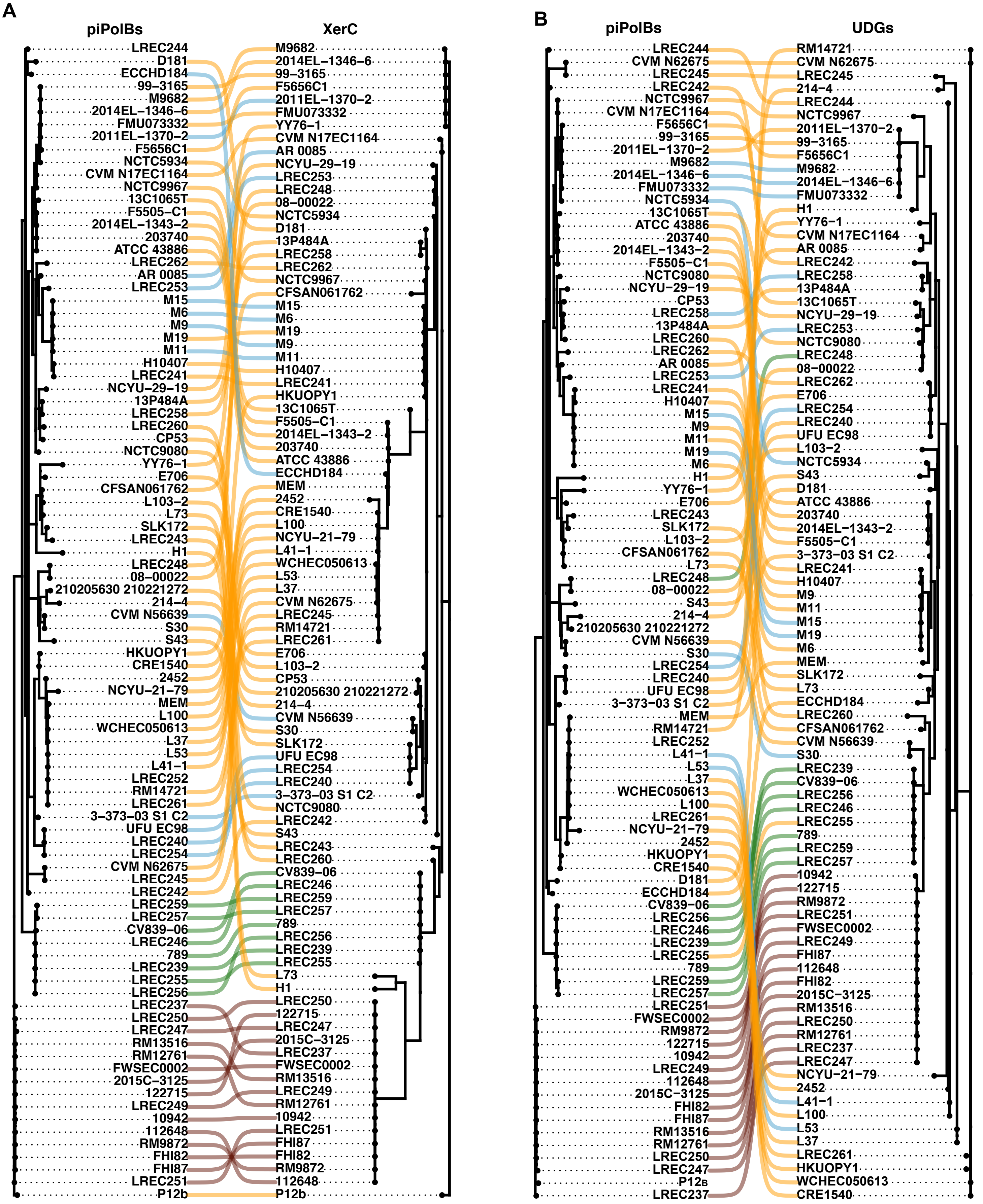
A
piPolBs
XerC
LREC244
D181
ECCHD184
99-3165
M9682
2014EL-1346-6
FMU073332
2011EL-1370-2
F5656C1
NCTC5934
CVM N17EC1164
NCTC9967
13C1065T
F5505-C1
2014EL-1343-2
203740
ATCC 43886
LREC262
AR 0085
LREC253
M15
M6
M9
M19
M11
H10407
LREC241
NCYU-29-19
13P484A
LREC258
LREC260
CP53
NCTC9080
YY76-1
E706
CFSAN061762
L103-2
L73
SLK172
LREC243
H1
LREC248
08-00022
210205630 210221272
214-4
CVM N56639
S30
S43
HKUOPY1
CRE1540
2452
NCYU-21-79
MEM
L100
WCHEC050613
L37
L53
L41-1
LREC252
RM14721
LREC261
3-373-03 S1 C2
UFU EC98
LREC240
LREC254
CVM N62675
LREC245
LREC242
LREC259
LREC257
CV839-06
LREC246
789
LREC239
LREC255
LREC256
LREC237
LREC250
LREC247
RM13516
RM12761
FWSEC0002
2015C-3125
122715
LREC249
10942
112648
RM9872
FHI82
FHI87
LREC251
P12b
M9682
2014EL-1346-6
99-3165
F5656C1
2011EL-1370-2
FMU073332
YY76-1
CVM N17EC1164
AR 0085
NCYU-29-19
LREC253
LREC248
08-00022
NCTC5934
D181
13P484A
LREC258
LREC262
NCTC9967
CFSAN061762
M15
M6
M19
M9
M11
H10407
LREC241
HKUOPY1
13C1065T
F5505-C1
2014EL-1343-2
203740
ATCC 43886
ECCHD184
MEM
2452
CRE1540
L100
NCYU-21-79
L41-1
WCHEC050613
L53
L37
CVM N62675
LREC245
RM14721
LREC261
E706
L103-2
CP53
210205630 210221272
214-4
CVM N56639
S30
SLK172
UFU EC98
LREC254
LREC240
3-373-03 S1 C2
NCTC9080
LREC242
S43
LREC243
LREC260
CV839-06
LREC246
LREC259
LREC257
789
LREC256
LREC239
LREC255
L73
H1
LREC250
122715
LREC247
2015C-3125
LREC237
FWSEC0002
RM13516
LREC249
RM12761
10942
LREC251
FHI87
FHI82
RM9872
112648
P12b
B
piPolBs
UDGs
LREC244
CVM N62675
LREC245
LREC242
NCTC9967
CVM N17EC1164
F5656C1
99-3165
2011EL-1370-2
M9682
2014EL-1346-6
FMU073332
NCTC5934
13C1065T
ATCC 43886
203740
2014EL-1343-2
F5505-C1
NCTC9080
NCYU-29-19
CP53
LREC258
13P484A
LREC260
LREC262
AR 0085
LREC253
LREC241
H10407
M15
M9
M11
M19
M6
H1
YY76-1
E706
LREC243
SLK172
L103-2
CFSAN061762
L73
LREC248
08-00022
S43
214-4
210205630 210221272
CVM N56639
S30
LREC254
LREC240
UFU EC98
3-373-03 S1 C2
MEM
RM14721
LREC252
L41-1
L53
L37
WCHEC050613
L100
LREC261
NCYU-21-79
2452
HKUOPY1
CRE1540
D181
ECCHD184
CV839-06
LREC256
LREC246
LREC239
LREC255
789
LREC259
LREC257
LREC251
FWSEC0002
RM9872
122715
10942
LREC249
112648
2015C-3125
FHI82
FHI87
RM13516
RM12761
LREC250
LREC247
P12B
LREC237
RM14721
CVM N62675
LREC245
214-4
LREC244
NCTC9967
2011EL-1370-2
99-3165
F5656C1
M9682
2014EL-1346-6
FMU073332
H1
YY76-1
CVM N17EC1164
AR 0085
LREC242
LREC258
13P484A
13C1065T
NCYU-29-19
LREC253
NCTC9080
LREC248
08-00022
LREC262
E706
LREC254
LREC240
UFU EC98
L103-2
NCTC5934
S43
D181
ATCC 43886
203740
2014EL-1343-2
F5505-C1
3-373-03 S1 C2
LREC241
H10407
M9
M11
M15
M19
M6
MEM
SLK172
L73
ECCHD184
LREC260
CFSAN061762
CVM N56639
S30
LREC239
CV839-06
LREC256
LREC246
LREC255
789
LREC259
LREC257
10942
122715
RM9872
LREC251
FWSEC0002
LREC249
FHI87
112648
FHI82
2015C-3125
RM13516
LREC250
RM12761
LREC237
LREC247
NCYU-21-79
2452
L41-1
L100
L53
L37
LREC261
HKUOPY1
WCHEC050613
CRE1540

Figure S6

**Figure S6. Tanglegram of pipolins and host strains.**
Tanglegram representation of maximum-likelihood comparative phylogenies of piPolB gene (same as in Figure S1) with XerC (A) and UDG (B) genes of pipolins. Association lines are colored based on the phylogenetic groups as in Figure S3.

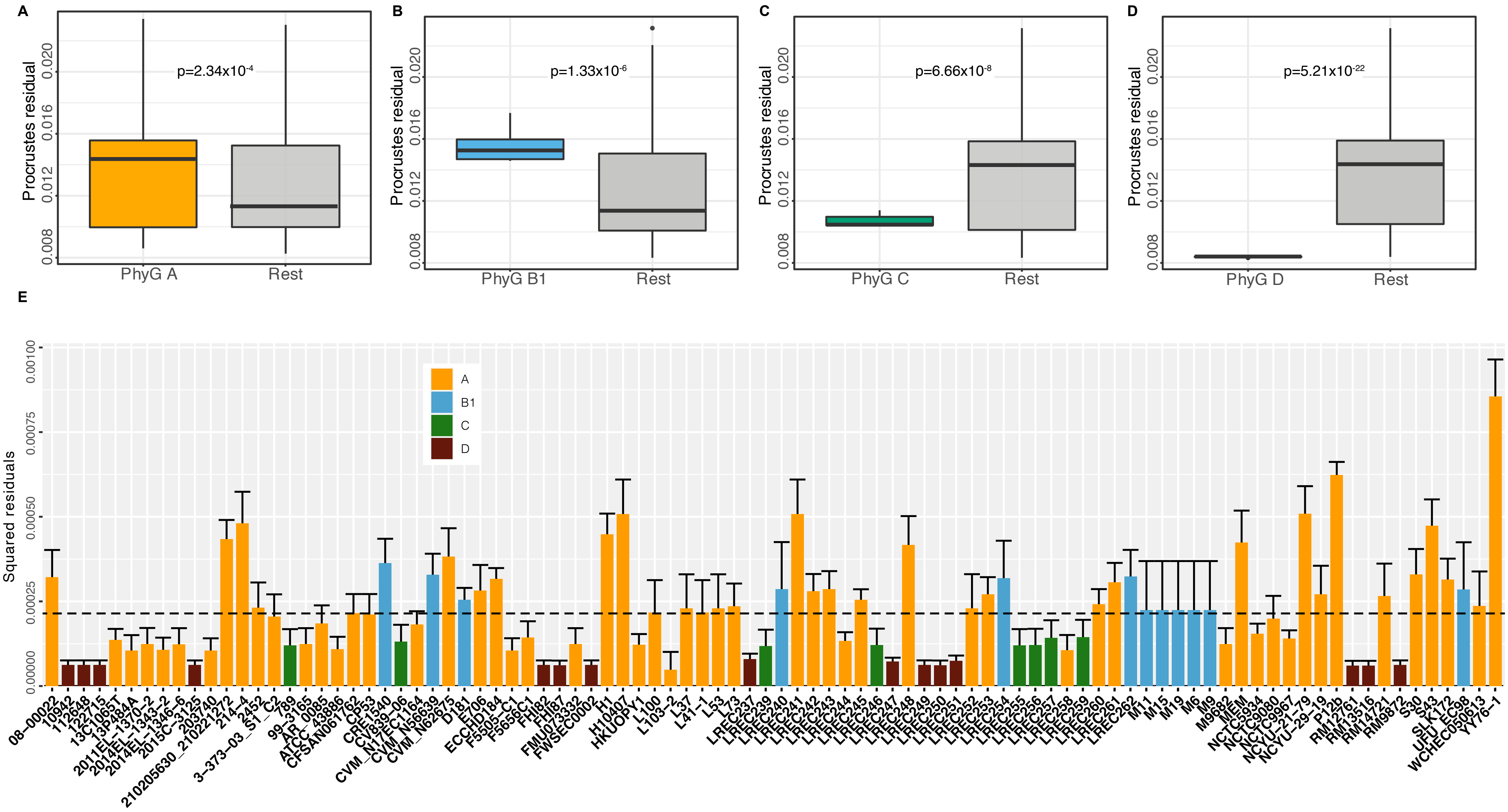

A
B
C
D
E
Procrustes residual
0.008
0.012
0.016
0.020
p=2.34x10-4
p=1.33x10-6
p=6.66x10-8
p=5.21x10-22
PhyG A
PhyG B1
PhyG C
PhyG D
Rest
Squared residuals
0.00000
0.00025
0.00050
0.00075
0.00100
A
B1
C
D
08−00022
10942
112648
122715
13C1065T
13P484A
2011EL−1370−2
2014EL−1343−2
2014EL−1346−6
2015C−3125
203740
210205630_210221272
214−4
2452
3−373−03_S1_C2
789
99−3165
AR_0085
ATCC_43886
CFSAN061762
CP53
CRE1540
CV839−06
CVM_N17EC1164
CVM_N56639
CVM_N62675
D181
E706
ECCHD184
F5505−C1
F5656C1
FHI82
FHI87
FMU073332
FWSEC0002
H1
H10407
HKUOPY1
L100
L103−2
L37
L41−1
L53
L73
LREC237
LREC239
LREC240
LREC241
LREC242
LREC243
LREC244
LREC245
LREC246
LREC247
LREC248
LREC249
LREC250
LREC251
LREC252
LREC253
LREC254
LREC255
LREC256
LREC257
LREC258
LREC259
LREC260
LREC261
LREC262
M11
M15
M19
M6
M9
M9682
MEM
NCTC5934
NCTC9080
NCTC9967
NCYU−21−79
NCYU−29−19
P12b
RM12761
RM13516
RM14721
RM9872
S30
S43
SLK172
UFU_EC98
WCHEC050613
YY76−1


Figure S7

**Figure S7. Detailed cophylogeny analysis of pipolins and host strains with PACo.** Squared Procrustes residues of each phylogenetic group were compared with the remainder interactions (A, B, C, D). The p-value of Welch t-test is indicated. Panel E shows the contribution of each pipolin-host association to the general pattern of coevolution. Each bar represents a jack-knifed estimate of a squared residual. Error bars represent the upper 95% confidence intervals from applying PACo to patristic distances. The dashed line indicates the median squared residual value that can serve as a threshold for congruent phylogenetic interactions. Bars are colored based on the phylogenetic groups of the strains.
