# Supplementary material for: High diversity and variability of pipolins among a wide range of pathogenic Escherichia coli strains
Source: Sci Rep. 2020 Jul 27;10:12452. doi: 10.1038/s41598-020-69356-6 (PMC7385651; doi:10.1038/s41598-020-69356-6)
Supplement: Supplementary file 4 — Supplementary Table S3 [file 41598_2020_69356_MOESM4_ESM.docx]

| **Gene** | **Pipolins (%)** | **Annotation** | **UniProtKB HHPred best hit** | **eggNOG Description** | **KEGG KO** | **KEGG Description** |
| --- | --- | --- | --- | --- | --- | --- |
| pipolB | 92 (100%) | Primer-independent DNA polymerase PolB | P03680 |  |  |  |
| xerC_2 | 90 (98%) | Tyrosine recombinase XerC | P0A8P8 | Belongs to the 'phage' integrase family |  |  |
| group_1 | 87 (95%) | Uracil-DNA glycosylase | Q96YD0 |  |  |  |
| group_6 | 86 (93%) | hypothetical protein |  |  |  |  |
| xerC_1 | 84 (85%) | Tyrosine recombinase XerC | P03700 | Belongs to the 'phage' integrase family |  |  |
| hisF | 78 (83%) | Type I restriction modification system methyltransferase (hsdM) | Q5M500 | HsdM N-terminal domain | [K03427](https://www.kegg.jp/dbget-bin/www_bget?ko:K03427) | hsdM; type I restriction enzyme M protein [EC:2.1.1.72] |
| group_16 | 76 (82%) | metallohydrolase | Q57587 | Metal-dependent hydrolase | [K07043](https://www.kegg.jp/dbget-bin/www_bget?ko:K07043) | uncharacterized protein |
| group_18 | 75 (82%) | hypothetical protein |  |  |  |  |
| group_5 | 75 (82%) | Type I site-specific deoxyribonuclease (hsdR) | P10486 | Type I restriction enzyme R protein N terminus (HSDR_N) | [K01153](https://www.kegg.jp/dbget-bin/www_bget?ko:K01153) | hsdR; type I restriction enzyme, R subunit [EC:3.1.21.3] |
| group_10 | 75 (82%) | Protein of unknown function (DUF2787) |  | Protein of unknown function (DUF2787) |  |  |
| group_13 | 74 (80%) | hypothetical protein |  | Protein of unknown function (DUF726) |  |  |
| group_11 | 73 (79%) | hypothetical protein |  |  |  |  |
| group_24 | 73 (79%) | hypothetical protein |  |  |  |  |
| group_52 | 73 (79%) | Excisionase | A6T888 |  |  |  |
| group_3 | 64 (70%) | hypothetical protein |  |  |  |  |
| group_8 | 59 (64%) | hypothetical protein |  |  |  |  |
| group_19 | 56 (61%) | WYL domain | A0A4Y3NDN0 | transcriptional regulator |  |  |
| group_58 | 53 (58%) | Znf/thioredoxin_put domain-containing protein | Q9A679 |  |  |  |
| group_28 | 41 (45%) | Uncharacterized protein family (UPF0149) | P28366 | Uncharacterised protein family (UPF0149) | [K07039](https://www.kegg.jp/dbget-bin/www_bget?ko:K07039) | uncharacterized protein |
| group_17 | 38 (41%) | IS1 family transposase IS1A |  | cog cog3677 |  |  |
| group_23 | 31 (34%) | PD-(D/E)XK nuclease superfamily |  | PD-(D/E)XK nuclease superfamily |  |  |
| group_31 | 30 (33%) | Restriction endonuclease | A0A0J9X157 | Restriction endonuclease |  |  |
| group_15 | 30 (33%) | IS1 family transposase IS1X2 |  | cog cog1662 | [K07480](https://www.kegg.jp/dbget-bin/www_bget?ko:K07480) | insertion element IS1 protein InsB |
| group_9 | 29 (32%) | Protein of unknown function (DUF4011) |  | Protein of unknown function (DUF4011) |  |  |
| group_34 | 24 (26%) | hypothetical protein |  | type I restriction enzyme, R |  |  |
| group_53 | 23 (25%) | Protein of unknown function DUF262 |  | Protein of unknown function (DUF1524) |  |  |
| group_14 | 22 (24%) | Uncharacterized protein family (UPF0149) |  |  |  |  |
| group_20 | 22 (24%) | WYL-domain containing protein | A0A4Y3NDN0 |  |  |  |
| group_39 | 22 (24%) | Protein of unknown function DUF262 |  | Protein of unknown function (DUF1524) |  |  |
| group_40 | 22 (24%) | hypothetical protein |  |  |  |  |
| group_25 | 22 (24%) | Protein of unknown function DUF262 |  | Protein of unknown function (DUF1524) |  |  |
| group_55 | 21 (23%) | Protein of unknown function DUF262 |  | Protein of unknown function DUF262 |  |  |
| group_32 | 19 (21%) | Znf/thioredoxin_put domain-containing protein | Q9A679 |  |  |  |
| group_27 | 16 (17%) | Type I restriction modification enzyme | Q8R9Q6 | Type I restriction modification DNA specificity domain | [K01154](https://www.kegg.jp/dbget-bin/www_bget?ko:K01154) | hsdS; type I restriction enzyme, S subunit [EC:3.1.21.3] |
| group_29 | 16 (17%) | IS3 family transposase ISEam1 | A0A0G3QIX7 | Transposase | [K07483](https://www.kegg.jp/dbget-bin/www_bget?ko:K07483) | transposase |
| insK | 78 (16%) | IS3 family transposase ISEc14 | T0PD67 | silverDB |  |  |

**Table S3. Functional characterization of the most common pipolin genes.**

Annotation of genes from Roary shell-genome (present in more than 15% of pipolins) is indicated. Functional groups in eggNOG and KEGG databases, as well as HHPred searches were also performed for a detailed functional characterization. See Methods for details.
